# Identification of Inflammation-Related Biomarkers in Diabetes of the Exocrine Pancreas With the Use of Weighted Gene Co-Expression Network Analysis

**DOI:** 10.3389/fendo.2022.839865

**Published:** 2022-04-14

**Authors:** Guoqing Li, Jinfang Sun, Jun Zhang, Yingqi Lv, Dechen Liu, Xiangyun Zhu, Liang Qi, Zhiwei Chen, Zheng Ye, Xianghui Su, Ling Li

**Affiliations:** ^1^ Department of Endocrinology, Zhongda Hospital, School of Medicine, Southeast University, Nanjing, China; ^2^ Key Laboratory of Environmental Medicine Engineering, Ministry of Education, School of Public Health, Southeast University, Nanjing, China; ^3^ Institute of Glucose and Lipid Metabolism, Southeast University, Nanjing, China; ^4^ Department of Clinical Science and Research, Zhongda Hospital, School of Medicine, Southeast University, Nanjing, China; ^5^ Department of Endocrinology, Hunan Provincial People’s Hospital, First Affiliated Hospital of Hunan Normal University, Hunan, China; ^6^ Department of Endocrinology, Changji Branch, First Affiliated Hospital of Xinjiang Medical University, Xinjiang, China

**Keywords:** weighted gene co-expression network analysis, hub gene, TLR4, miRNAs–mRNAs, diabetes of the exocrine pancreas

## Abstract

Diabetes of the exocrine pancreas (DEP), also commonly described as pancreatogenic diabetes mellitus, is a type of diabetes secondary to abnormalities in pancreatic or exocrine secretion of the pancreas. However, its pathogenesis is not yet known. The aim of this article was to explore the biomarkers of DEP and their potential molecular mechanisms. Based on GSE76896 dataset, which was acquired from Gene Expression Omnibus (GEO), we identified 373 genes by weighted gene co-expression network analysis (WGCNA) and differential expression analysis. In addition, protein–protein interaction (PPI) network analysis and cytoHubba were used to screen potential hub genes. Five hub genes were determined, comprising Toll-like receptor 4 (TLR4), ITGAM, ITGB2, PTPRC, and CSF1R. Gene Ontology (GO) analysis and Kyoto Encyclopedia of Genes and Genomes (KEGG) pathways suggested macrophage activation and Toll-like receptor signaling pathway as important pathophysiological features of DEP. CIBERSORT suggested that TLR4 may regulate the immune pathway via macrophages. Next, we validated the expression and receiver operating characteristic curve (ROC) of the hub genes using the GSE164416 dataset. In addition, we used miRNet to predict the target miRNAs of hub genes and intersected them with common miRNAs in diabetes from the Human MicroRNA Disease Database (HMDD), which was used to propose a possible mechanistic model for DEP. The miRNA–mRNA network showed that has-miR-155-5p/has-miR-27a-3p/has-miR-21-5p-TLR4 might lead to TLR4 signaling pathway activation in DEP. In conclusion, we identified five hub genes, namely, TLR4, ITGAM, ITGB2, PTPRC, and CSF1R, as biomarkers to aid in the diagnosis of DEP and conducted an in-depth study of the pathogenesis of DEP at the genetic level.

## Introduction

Diabetes of the exocrine pancreas (DEP), also commonly described as pancreatogenic diabetes mellitus, is a type of diabetes secondary to abnormalities in pancreatic or exocrine secretion of the pancreas and currently accounts for approximately 8% of all diabetes mellitus (DM) (1, 2). Unlike type 2 (T2DM) and type 1 diabetes (T1DM), DEP is still poorly understood clinically. However, recent reports suggest that DEP may be more prevalent than commonly believed. It has also been shown that this clinically important disease may have been misdiagnosed due to inadequate awareness and screening difficulties (3, 4). Its etiology includes acute and chronic pancreatitis, pancreatic trauma, pancreatic tumors, cystic fibrosis of the pancreas, and other exocrine pancreatic disorders (5). Most of the current studies have focused on retrospective clinical studies, but the specific pathogenesis of DEP remains unclear.

Various pathologies such as pancreatitis and pancreatic cancer lead to changes in the islet microenvironment, like inflammation and fibrosis of the islets, which in turn affects endocrine function. Islets are distributed in the pancreatic alveoli, which are scattered oval or spherical clusters of cells, accounting for only 1%–2% of the mass of pancreas (6). Islets are made up of endocrine cells, vascular cells, and immune cells immersed in the extracellular matrix (7). Abnormal glucose metabolism occurs when insulin secretion is reduced after beta cell damage. In addition, the alpha cells of DEP patients are also damaged, and the ability of the pancreas to regulate hypoglycemia is severely limited, making them prone to hypoglycemia (8, 9). Immune cells also play an important role in the islet microenvironment of diabetic patients. Previous studies reporting about the role of immune cells located in the islet microenvironment have focused on T1DM, due to autoimmune reactions leading to β-cell damage (10). However, recent studies have suggested that macrophages play an essential role in influencing β-cell function (11). So far, the role of macrophages in DEP remains unclear.

**Figure 1:**
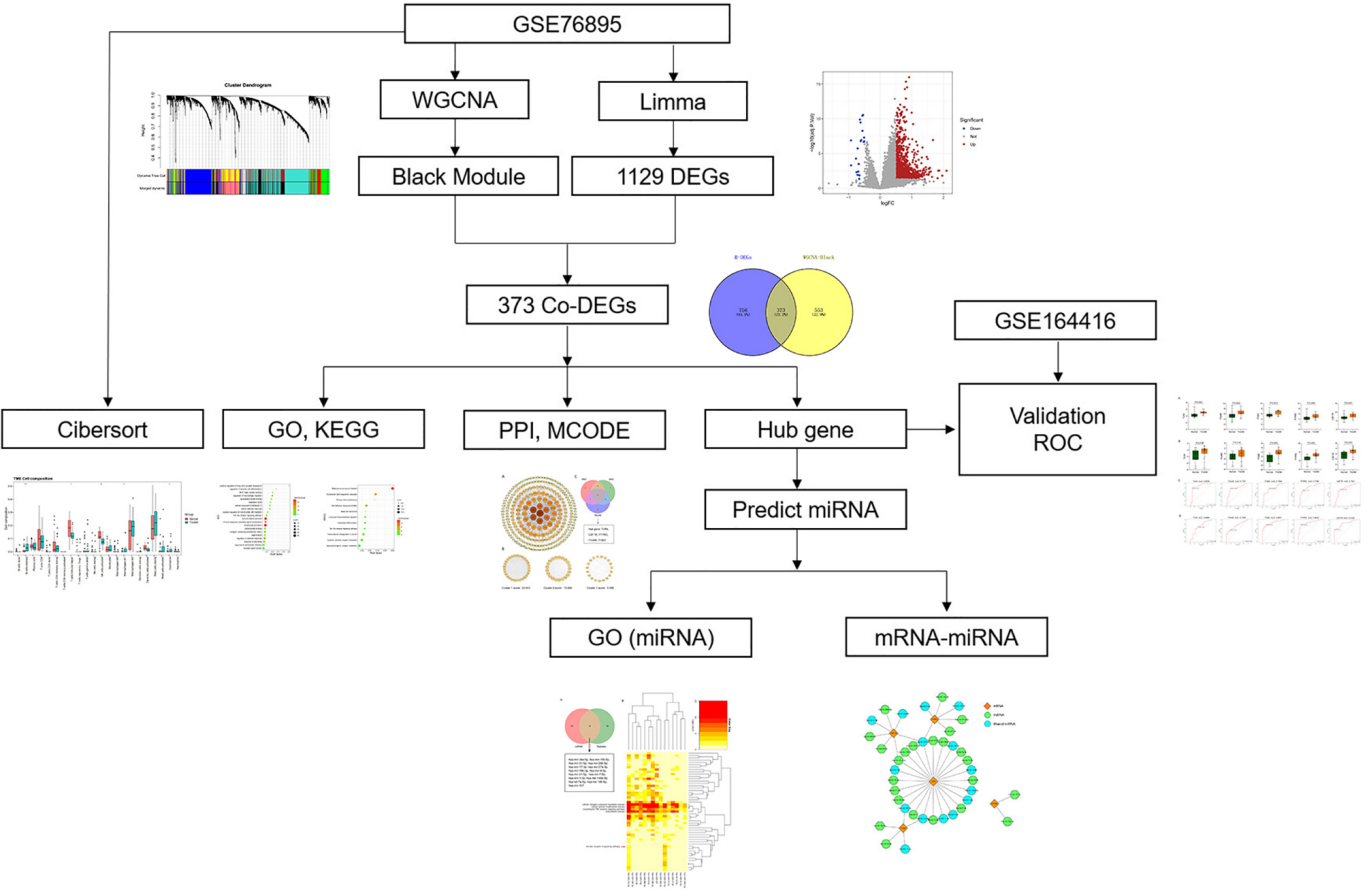
Research flowchart.

With the fast evolution of bioinformatics, a substantial amount of transcriptomics and microarrays can be used to find pivotal genes, signaling pathways of diseases to understand the pathogenesis and provide therapeutic approaches. Weighted gene co-expression network analysis (WGCNA), one of the most common analytical tools in bioinformatics, is often applied to study the associations between gene expression and clinical traits (12). Herein, we collected DEP microarray datasets from the Gene Expression Omnibus (GEO) and screened out hub genes via combined WGCNA, differentially expressed gene (DEG), and validation in a separate dataset. Then, we assessed immune cell infiltration in DEP by Cell-Type Identification by Estimating Relative Subsets of RNA Transcripts (CIBERSORT). Finally, according to predicted results of microRNAs (miRNAs), we constructed miRNA–mRNA network to gain insight into the pathogenesis of DEP. To our knowledge, this might be the first research to explore the pathogenesis of DEP using a combination of WGCNA and epigenetics. The research flow of this paper is shown in **Figure 1**.

## Methods

### Microarray Data Acquisition and Analysis

The gene matrix for DEP was obtained from the GEO database. Screening was according to the following criteria: (1) the gene sets must include experimental and control groups, (2) DEP patients, and (3) the samples of datasets more than 10. Finally, GPL570 dataset GSE76896, which included 32 non-diabetic samples and 20 DEP samples, was selected as the test set; GPL16791 dataset GSE164416, which included 18 non-diabetic samples and 35 DEP samples, was selected as validation set. In addition, DEP patients were all from metabolically phenotyped pancreatectomized patients with < 1 year duration of diabetes, and their general information is presented in **Supplementary Table S1**. We normalized the GSE76896 microarray using the robust multiarray averaging (RMA) algorithm. The “limma” package in the R software was used to screen the DEGs in GSE76896 (13). The parameters were set to adj.P.Val < 0.05 and |log2FC| ≥ 0.5.

### Construction of WGCNA

WGCNA is a common method for systems biology analysis, which identifies key genes by linking gene networks to clinical traits. The top 25% of genes with the largest variance in all samples were selected as the input dataset for the subsequent WGCNA. First, to guarantee that the constructed network was valid and reliable, we removed the outlier cases by hierarchical clustering analysis. Then, the appropriate soft threshold was determined by scale independence and mean connectivity, and the correlation matrix was converted into an adjacency matrix. Subsequently, the adjacency matrix was further processed into a topological overlap matrix (TOM). The dynamic tree cutting method was performed by hierarchical clustering of genes to identify modules. The minimum size cutoff of 30 and a deepSplit value of 2 were chosen as the distance measure for the dendrogram. Eigengene was calculated to merge comparable modules. Finally, the WGCNA package was used for analysis and preservation (12).

### Selecting Meaningful Modules

To select meaningful modules, Pearson’s correlation test was applied to evaluate the correlation between features and gene modules. Then, we defined the module–gene correlation as module membership (MM) and the trait–gene correlation as the gene significance (GS). Finally, we further analyzed the correlation between MM and GS in the selected meaningful modules.

### Identification of Gene Clusters and Hub Genes

The intersection of the DEGs was taken with the genes in our selected module and as a new set of genes. The results were visualized by using Venn diagram. The PPI network according to all Co-DEGs was built through the online STRING. Next, we imported the interaction results into Cytoscape software (v3.7.2) to construct the PPI network for better visualization. Then, the Minimal Common Oncology Data Elements (MCODE) was performed to extract significant gene clusters and acquire cluster scores (screening criteria: max depth=100; k-core=2; node score cutoff=0.2; degree cutoff=2). Gene clusters with scores >5 were focused. In addition, hub genes in the network was identified by cytoHubba (14). Three algorithms were used, namely, Maximal Clique Centrality (MCC), Maximum Neighborhood Component (MNC), and Degree, to identify the top 10 core genes. Finally, the results of the three algorithms were crossed to get the final hub genes.

### Functional Enrichment Analysis

To further explore the characteristics and functions of Co-DEGs, we used Gene Ontology (GO) annotations to characterize biological properties, including molecular function, cellular component, and biological process (15). Kyoto Encyclopedia of Genes and Genomes (KEGG) pathway enrichment analysis was conducted to determine functional attributes (16). The threshold for significant terms was set as p < 0.01.

### CIBERSORT Analysis

CIBERSORT is an important tool for measuring immune infiltration and can be utilized to assess the abundance of member cell types in mixed cell populations based on gene expression (17). Immune cell signatures for each sample were obtained from GSE76896 using CIBERSORT with the number of permutations set to 100. Wilcoxon rank-sum test was then used to compare the proportion of islet immune cells in the normal and DEP groups, and the result was presented in a box plot.

### Validation of Hub Genes

The hub genes that have not been studied in DEP were further validated in another dataset GSE164416 downloaded from the GEO database. In the GSE164416 dataset, “EdgeR” was used for linear correction of expression profiles. Then, the corrected gene matrix was extracted by “Limma Voom.” In addition, ROC curves for selected genes were calculated using “pROC” package *via* R software to assess the ability of hub genes to diagnose DEP.

### Identification of the Common MicroRNAs in DM

miRNAs are small non-coding RNAs that have been shown to regulate gene expression by inhibiting mRNA translation or promoting mRNA degradation (18). Therefore, miRNet (version 2.0) was used to predict target miRNAs of hub genes. The Human microRNA Disease Database (HMDD) is a database that collects experimental evidence for the associations of human miRNA with disease (19). We acquired DM-associated miRNAs and intersected with miRNAs predicted by hub genes. To explore the function of miRNAs, GO BP analysis was performed using mirPath v3.0 software in DIANA Tools (20). A p-value <0.01 was used to screen for significant GO terms. The miRNA–mRNA interaction network according to the association between miRNAs and mRNAs was constructed by using Cytoscape.

### Statistics Analysis

The R software was used for statistical analysis, and the ggplot2 package was used for box plots. Data were analyzed using SPSS 23.0, and Student’s t-test or Wilcoxon rank-sum test was performed to compare the differences between the two groups. A two-tailed p < 0.05 was assumed statistically significant.

## Results

### Construction of WGCNA

**Figure 2:**
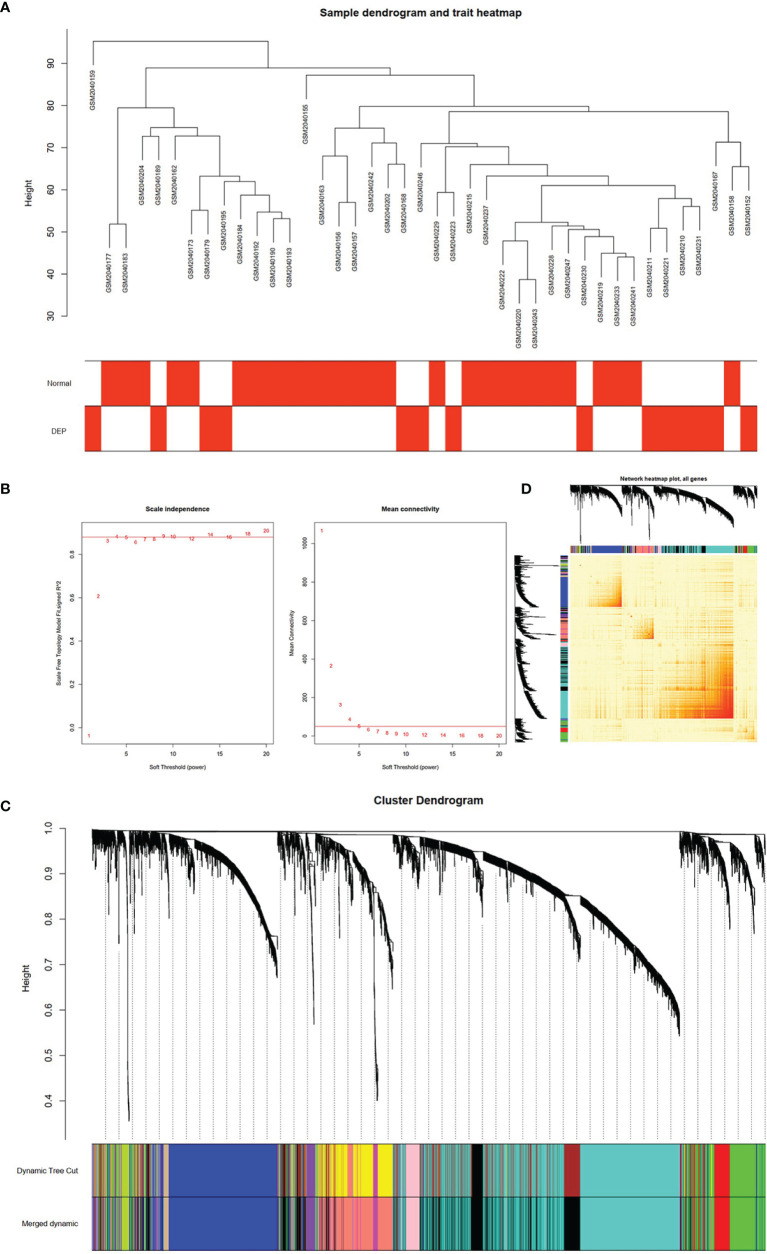
Construction of WGCNA model for GSE76896 dataset. **(A)** Sample dendrogram of two groups. **(B)** The value of scale independence on the left and the value of mean connectivity on the right. **(C)** The cluster dendrogram of all genes, according to the 1-TOM matrix, were grouped into different modules. Each color represents a module. **(D)** Network heat map of all genes (a color change from yellow to red indicates a high degree of overlap between modules).

After matching the sample traits with the expression matrix, the sample cluster tree is shown in **Figure 2A**; meanwhile, 5 was selected as the soft-threshold power according to the scale independence and mean connectivity values (**Figure 2B**), which indicates that a scale-free network has been successfully constructed. A total of 12 modules were divided by WGCNA, which were indicated by different colors, and the larger the area of the module, the higher the number of genes included (**Figure 2C**). In addition, based on TOM, the network heat map plot between all genes is shown in **Figure 2D**, indicating that these modules were independent of each other (**Figure 2D**).

### Analysis of Module–Trait Relationship

**Figure 3:**
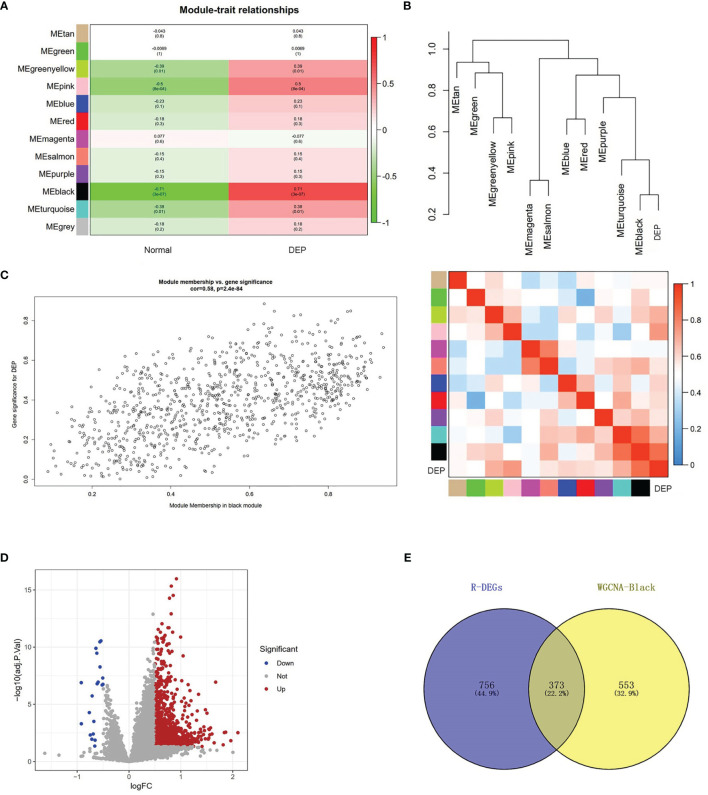
Module–trait correlations analysis and screening of DEGs. **(A)** The relationship between modules and trait. There are 12 modules and two traits in total. Correlation coefficients and corresponding p value are listed for each cell. **(B)** The cluster diagram of each module and DEP (above) and the heat map of DEP and each module (below). **(C)** The genes in the black module were significantly correlated with DEP. **(D)** Volcano plot showing the differential genes in the GSE76896 dataset. **(E)** The Venn diagram illustrates Co-DEGs between the black module and DEGs.

The relationship between modules and trait indicated that several modules were associated with DEP, and it clearly showed that the black module (r=0.71, p=3e−07) was most significantly associated with DEP (**Figure 3A**). In addition, DEP was combined with modules to redo the clustering tree and to make a heat map of Mes correlations. The shades of the overlapping colors of the heat map suggested the magnitude of the correlation between the different modules (**Figure 3B**). Moreover, **Figure 3C** shows that these genes in the black module had a high correlation with DEP, and the different modules were independent of each other (cor=0.58, p=2.4e−84) (**Figure 3C**).

### Screening of the Co-DEGs

To further identify genes with differences in the black module, we used the limma package processing dataset GSE76896 to identify DEGs. Compared with non-diabetic samples, a total of 1,129 DEGs were identified in the DEP samples; volcano plot was used to visualize these DEGs (**Figure 3D**). The top 20 upregulated genes and 10 downregulated genes identified in the DEGs are shown in **Supplementary Table S2**. Next, The Co-DEGs were obtained from the intersection of the black module and the DEGs, including 373 genes, and the result is shown in **Figure 3E**.

### Identification of Gene Clusters and Hub Genes

**Figure 4:**
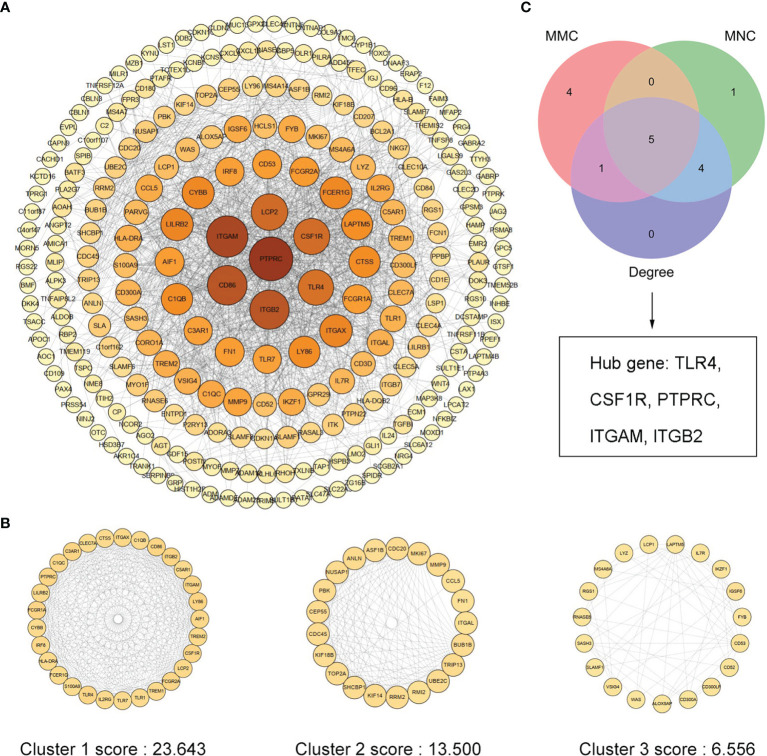
PPI network of Co-DEGs and screening of hub genes. **(A)** Cytoscape embellishes the results of the interactions between the proteins encoded by Co-DEGs, consisting of 255 nodes and 1,636 edges. Different nodes represent different proteins, and each edge indicates one protein–protein interaction. The value of the gene degree is represented by the size of the node. **(B)** A total of three cluster modules identified by MCODE, all score >5. **(C)** The Venn diagram illustrates five hub genes screened by three algorithms.

We uploaded 373 Co-DeGs to STRING for PPI analysis, resulting in a total of 255 nodes and 1,636 edges. The result of interactions was then further analyzed using Cytoscape as shown in **Figure 4A**. Moreover, the color shades of the node indicated its level of neighborhood connectivity, and the magnitude of the node indicated the value of degree. Network data was processed with MCODE to identify gene clusters, and clusters with scores >5 were focused. A total of three gene clusters with high scores were screened, and their scores were 23.643, 13.500, and 6.556, with cluster 1 being the most important (**Figure 4B**). We used the cytoHubba in Cytoscape to determine hub genes and identified five hub genes by crossing the results of the three algorithms, including Degree, MCC, and MNC (**Figure 4C**). Details of the five hub genes are shown in **Table 1**.

**Table 1:** Five hub genes screened by three algorithms of cytoHubba.

Gene	Description	log2FC	adj.P.Val	Regulation
ITGB2	Integrin subunit beta 2	0.769	0.0085	Up
PTPRC	Protein tyrosine phosphatase Receptor type C	0.765	0.0218	Up
TLR4	Toll-like receptor 4	0.737	0.0011	Up
ITGAM	Integrin subunit alpha M	1.217	0.0093	Up
CSF1R	Colony stimulating factor 1 receptor	1.167	0.0136	Up

FC, fold change.

### The Unique Gene Signatures in DEP

**Figure 5:**
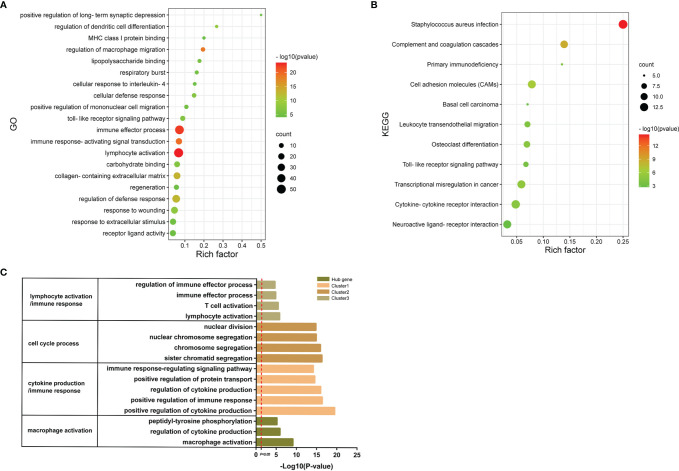
GO annotations and KEGG. **(A)** The bubble diagram shows the results of GO annotations of Co-DEGs. The color shades represent the level of p-value, while the size of the bubbles indicates the number of genes. **(B)** The bubble diagram illustrates the results of KEGG pathway of Co-DEGs. The color shades represent the level of p-value, while the size of the bubbles indicates the number of genes. **(C)** Results of GO annotations of clusters and hub genes.

In order to explore the signatures of Co-DEGs, we performed GO annotation and KEGG pathway analyses (**Figures 5A, B**). The results of GO annotation suggested that these differential genes were mostly enriched in processes involved in immune effector process, regulation of macrophage migration, and toll-like receptor signaling pathway. Considering the KEGG pathway, the majority of genes were enriched in cell adhesion molecules (CAMs), cytokine–cytokine receptor interaction, and Toll-like receptor signaling pathway. For each gene cluster and hub genes, we chose several keywords to describe their major biological functions. Among them, hub genes were mainly related to macrophage activation (**Figure 5C**).

### Performance of CIBERSORT

**Figure 6:**
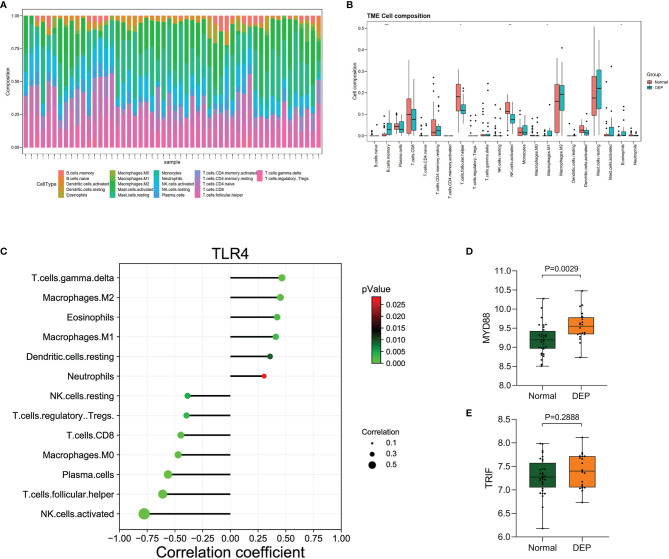
Immune cell infiltration analysis by CIBERSORT. **(A)** The percentage of 22 types of immune cells in all samples **(B)**. Box plot illustrates the distribution of 22 types of immune cells in the control group and DEP patients in GSE76896. p < 0.05 was considered statistically significant. *p < 0.05, **p < 0.01, ***p < 0.001. **(C)** The relationship between TLR4 and immune cell infiltration level. **(D, E)** The expression of MYD88 and TRIF in GSE76896.

We detected the infiltration of immune cells in the islet microenvironment by CIBERSORT. The bar chart showed the proportion of immune cells in all samples (**Figure 6A**). Obviously, macrophages accounted for the majority of all infiltrating cells. Then, we compared the differences in immune-infiltrating cells between the normal group and the DEP group (**Figure 6B**). Five types of immune cells, namely, memory B cells, M1 macrophages, eosinophils, follicular helper T (Tfh) cells, and activated natural killer (NK) cells, were differentially expressed (all p<0.05). We focused on the correlation between TLR4 and immune-infiltrating cells (**Figure 6C**). Among them, six immune cells were positively correlated with TLR4, and seven immune cells were negatively correlated with TLR4. Also based on the GSE76896 dataset, we found that MYD88 expression was elevated in DEP (p<0.05), while TRIF expression was unchanged (**Figures 6D, E**). What is more, we detected the correlation between several other hub genes and immune-infiltrating cells, and both of them were positively correlated with M1 macrophages (**Supplementary Figure S1**).

### Validation of Hub Genes

**Figure 7:**
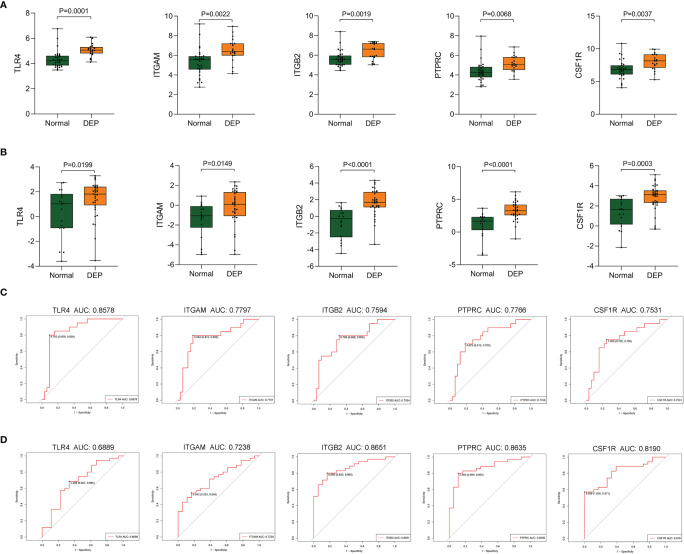
Validation of hub genes. **(A, B)** The expression of hub genes including TLR4, ITGAM, ITGB2, PTPRC, and CSF1R was detected in GSE76896 and GSE164416. **(C, D)** ROC curve of the selected genes in GSE76896 and GSE164416.

To verify screened genes, we analyzed the expression of hub genes in the GSE164416 dataset. As shown in **Figure 7**, the expression of TLR4, ITGAM, ITGB2, PTPRC, and CSF1R was significantly upregulated (all p < 0.05) in the islets of DEP compared to the control group in datasets GSE76896 and GSE164416 (**Figures 7A, B**). What is more, we plotted the ROC curves of hub genes and calculated the area under the curve (AUC) to distinguish DEP from the control group. Among the five hub genes, TLR4 has the highest diagnostic value. The diagnostic values of hub genes in GSE76896 are follows: TLR4 (AUC, 0.8578), ITGAM (AUC, 0.7797), PTPRC (AUC, 0.7766), ITGB2 (AUC, 0.7594), and CSF1R (AUC, 0.7531) (**Figure 7C**). In addition, they also have high diagnostic value in the GSE164416 dataset (**Figure 7D**).

### Identified and Analysis of Common miRNAs in DM

**Figure 8:**
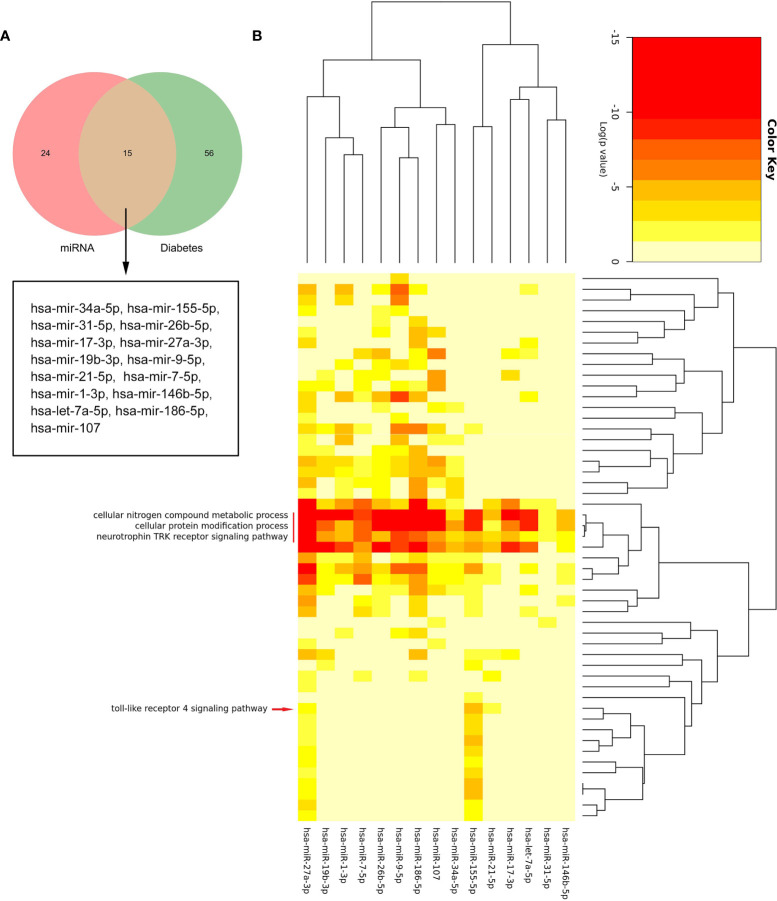
The biological process analysis of the common miRNAs. **(A)** The Venn diagram of common miRNAs between predicted miRNAs and Human microRNA Disease Database (HMDD). **(B)** The red arrow indicated the toll-like receptor 4 signaling pathway. The red line represents the GO BP regulated by common miRNAs.

By searching the HMDD database, we found that 71 miRNAs were related to DM (**Supplementary Table S3**). **Figure 8A** shows that there were 15 common miRNAs in diabetes-related and predicted miRNAs (**Figure 8A**). Then, the signatures of these 15 miRNAs were investigated. The enrichment analysis revealed that three biological processes, namely, cellular nitrogen compound metabolic process, cellular protein modification process, and neurotrophin TRK receptor signaling pathway, were regulated by all 15 miRNAs. Interestingly, these results also included “toll-like receptor 4 signaling pathway,” which suggested that those common miRNAs involved in DM could also regulate the TLR4 signaling pathway (**Figure 8B**). This confirmed our analysis again.

### Construction of miRNA–mRNA Networks

**Figure 9:**
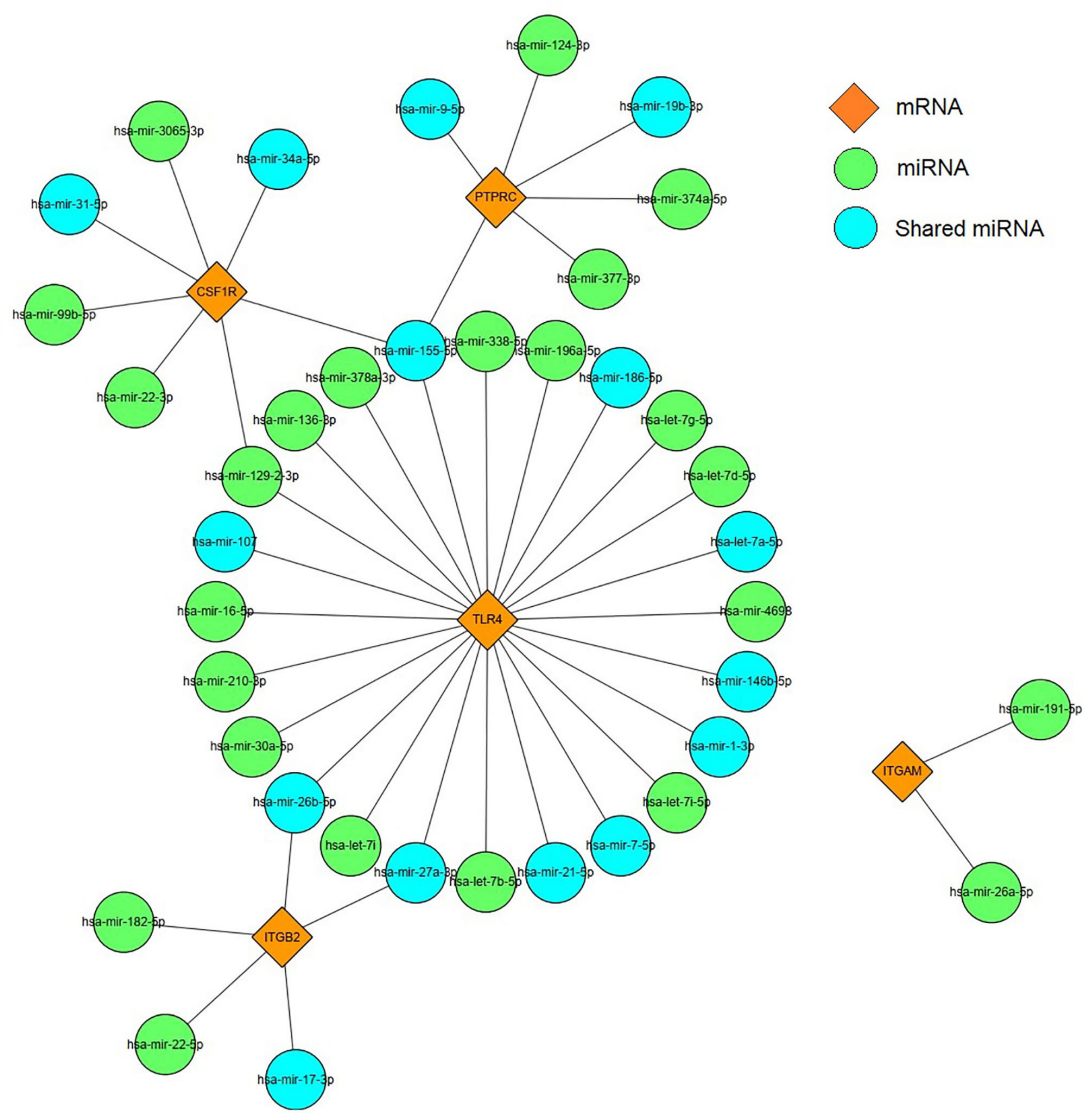
miRNAs–mRNA regulatory network. Orange diamonds show the core genes; green circles show predicted miRNAs. Blue circles represent common miRNAs.

Based on the prediction results of miRNet database, an miRNA and mRNA co-expression regulatory network was constructed by Cytoscape, which consisted of 44 nodes and 44 edges (**Figure 9**). Among these miRNAs, the blue color represents the miRNA associated with DM. Combined with the enrichment results of miRNA, TLR4 signaling pathway may be regulated by has-miR-155-5p, has-miR-27a-3p, and has-miR-21-5p. Thus, we hypothesized that has-miR-155-5p/has-miR-27a-3p/has-miR-21-5p-TLR4 might lead to TLR4 signaling pathway activation in DEP.

## Discussion

The pathophysiological mechanism of DEP is mainly caused by pancreatic inflammation and fibrosis of the islets, which ultimately leads to loss of the islets (21). The relative deficiency of insulin, glucagon, and pancreatic polypeptides may lead to a high degree of glycemic variability (22). Early diagnosis and treatment can prevent complications. Therefore, it is essential to identify effective biomarkers and explore the pathogenesis of DEP. For the first time, we investigated the pathogenesis of DEP using the WGCNA, which could obtain meaningful modules that screen for genes associated with clinical traits.

In the present study, we identified 1,129 DEGs in DEP and control groups. Furthermore, a total of 12 co-expression modules were acquired according to WGCNA analysis. Among them, the black module had the highest correlation with DEP, including 373 Co-DEGs. Moreover, there were modules such as pink involved in DEP. Therefore, DEP involves a complex network of gene regulation. Then, we constructed a PPI network using Cytoscape; three gene clusters were found with scores >5. We identified five hub genes, comprising TLR4, ITGAM, ITGB2, PTPRC, and CSF1R, based on the three algorithms of cytoHubba. GO and KEGG analyses suggested that many of these Co-DEGs were involved in immune effector process, and they all indicated Toll-like receptor signaling pathway activation. Moreover, we performed GO analysis of five hub genes. The results suggested that macrophage activation plays an important role in the pathogenesis of DEP. Indeed, all five hub genes are important markers of macrophages. Among them, TLR4 is a classical marker of M1 macrophage polarization (23). CSF1R is an important pathway for the polarization of M2 macrophages (24). In addition, the upregulated expression of PTPRC, ITGAM, and ITGB2 in macrophages is also associated with inflammation (25–27). Our results suggested that the expression levels of hub genes were all elevated in DEP compared to controls, and the ROC curves indicated that TLR4 had a high diagnostic value. Therefore, we focused on the role of TLR4 in DEP.

**Figure 10:**
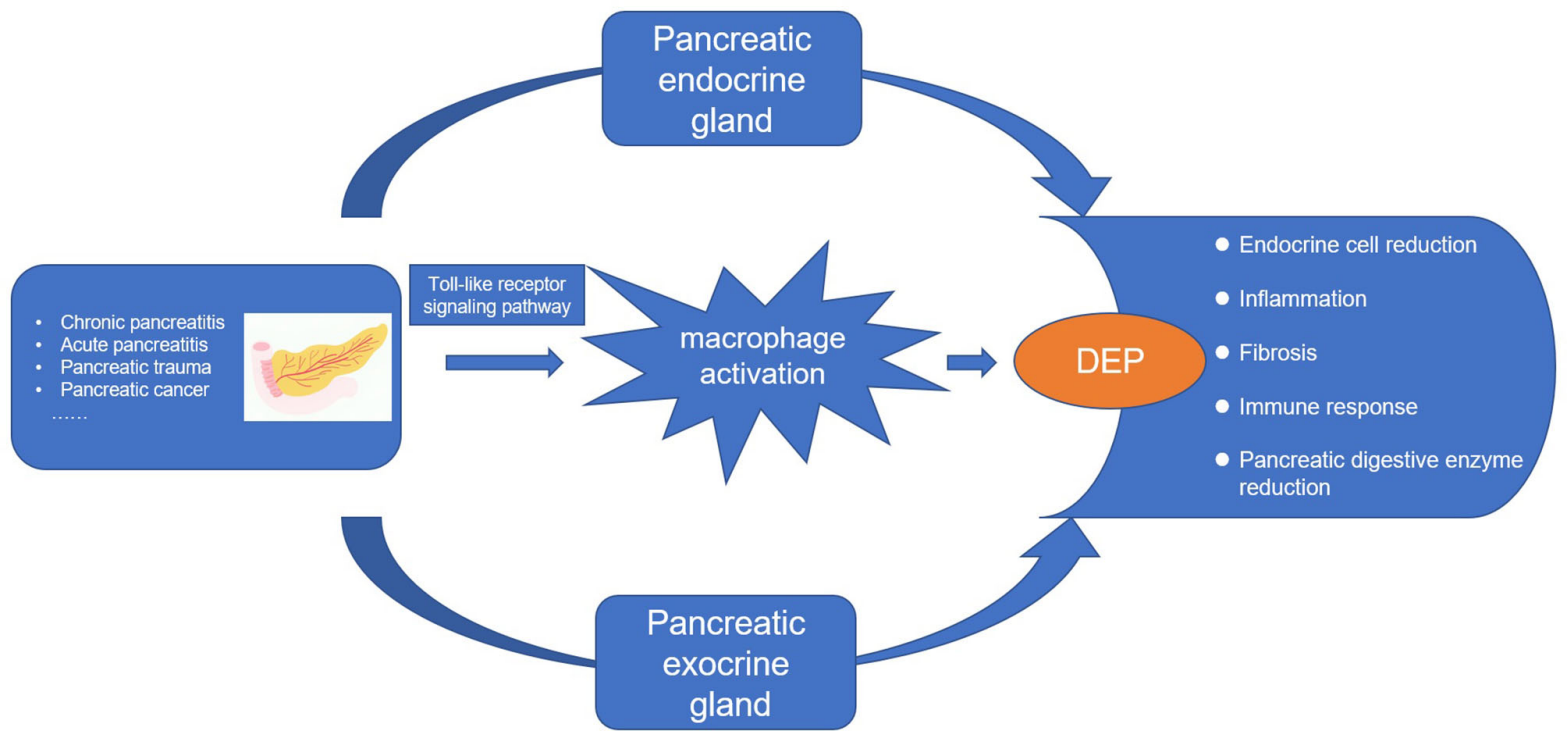
The disease road model. Macrophage activation plays an important role in the pathogenesis of DEP. Various pancreatic diseases lead to the development of DEP through the endocrine and exocrine glands of the pancreas. Toll-like receptor 4 signaling pathway induces macrophage activation and secretes proinflammatory factors, which is an important pathogenesis.

Whether it is pancreatitis or pancreatic cancer, the underlying immune mechanisms play an important role in the pathogenesis of DEP (21). Studies have shown that inflammatory cytokines such as interleukin (IL)-1β and interferon gamma (IFNγ) can inhibit glucose-stimulated insulin secretion (28). In addition, the latest longitudinal study found that elevated plasma levels of IL-1β and IFNγ at baseline were significant predictors of the development of new-onset prediabetes after acute pancreatitis (NOPAP) during follow-up (29). We likewise found the immune effector process in DEP. Studies have shown that the immune cells in islets are mainly macrophages, in addition to a small number of T cells, B cells, and NK cells (30, 31). Indeed, we found by using CIBERSORT that macrophages were the majority of the infiltrating cells. Our study also analyzed the differences in immune-infiltrating cells between the islets of DEP and normal samples. There were no changes in CD4 T cells and CD8 T cells between the two groups, which also ruled out the possibility of T1DM. A total of five immune cell proportions differed between DEP and normal groups. It is worth noting that M1 macrophages were significantly increased in DEP, which was consistent with our finding of macrophage activation. Macrophages are usually widely divided into M1 and M2, where M1 macrophages have proinflammatory effects associated with the immune response of bacteria and intracellular pathogens, while M2 macrophages have anti-inflammatory effects and play a role in angiogenesis and wound healing (32). The inflammatory environment can initiate the conversion of islet macrophages into M1 macrophages. When M1 macrophages are activated, they usually secrete large amounts of cytokines such as IL6 and tumor necrosis factor alpha (TNF-a) (33). So far, some cross-sectional studies have shown that serum levels of IL6 and TNF-a are elevated after acute pancreatitis and are associated with chronic hyperglycemia after pancreatitis (CHAP) (34, 35). Therefore, we proposed that M1 macrophages also play an important role in DEP. In addition, we analyzed the relationship between hub genes and immune-infiltrating cells. Among them, the expression of TLR4 was positively correlated with M1 macrophages. TLR4 is closely associated with β-cell dysfunction. Manesh et al. found that TLR4 co-localized with islet endothelial cells and macrophages but mainly mediated cytokine secretion through macrophages (36). TLR4 signaling pathway usually includes MyD88-dependent and MyD88-independent pathways (37). We examined the expression of MYD88 and TRIF in the GSE76896 dataset. MYD88 was significantly elevated in DEP compared to controls, while TRIF was unchanged. Therefore, we hypothesized that in DEP, the TLR4 signaling pathway activates M1 macrophages mainly through the classical MyD88-dependent pathway, which mediates the release of inflammatory factors. As shown in **Figure 10**, based on the results of our analysis and existing theories, we presented a disease pathway model to account for the possible pathogenesis of DEP.

miRNAs have been widely confirmed to be connected with the development of various diseases, and they can reduce protein translation by binding to mRNAs; in addition, there are also a few reports that miRNAs can upregulate translation levels (18, 38). Therefore, we used the HMDD database and miRNet to construct miRNA–mRNA network. We performed a functional analysis of the common miRNAs. Interestingly, the GO analysis results still included toll-like receptor 4 signaling pathway. Among them, mainly has-miR-155-5p, has-miR-27a-3p, and has-miR-21-5p played a regulatory role. Then, we conducted a literature search. Studies have reported that has-miR-155-5p negatively regulates the level of SOCS-1, which is a key component of TLR4 signaling regulation (39). In addition, it has been demonstrated that has-miR-27a-3p and has-miR-21-5p can reduce inflammatory responses by inhibiting TLR4 expression (40, 41). Due to the critical role of has-miR-155-5p, has-miR-27a-3p, and has-miR-21-5p in regulating the TLR4 signaling pathway, they might be potential targets for the treatment of DEP.

The present study had several limitations. First, our sample size was small, and five hub genes as potential biomarkers need to be further validated in a multicenter, large sample population. Second, we did not validate the expression of hub genes in external experiments because islets are more difficult to obtain in DEP patients. Finally, the results of immune cells obtained using CIBERSORT calculations were relative expressions, which is somewhat inadequate to characterize the islet microenvironment. Therefore, we need more experiments such as immunohistochemistry and flow cytometry to validate it in the future.

## Conclusion

Our work identified five hub genes, namely, TLR4, ITGAM, ITGB2, PTPRC, and CSF1R, as potential biomarkers for DEP; meanwhile, we found that TLR4-mediated macrophage activation plays an important role in the pathogenesis of DEP. In addition, we also hypothesized that has-miR-155-5p/has-miR-27a-3p/has-miR-21-5p-TLR4 could affect the TLR4 signaling pathway in DEP. Thus, we provided clues to the mechanism of DEP by combining transcriptomics and epigenetics.

## Data Availability Statement

The datasets presented in this study can be found in online repositories. The names of the repository/repositories and accession number(s) can be found in the article/**Supplementary Material**.

## Author Contributions

Conceptualization, LL and XS. Data acquisition and processing, GL and JS. Interpretation of data, GL and JZ. Software, YL and ZY. Validation, DL, XZ, ZC, and LQ. GL and JS were responsible for writing the initial article. Revision and finalization, LL and XS. All authors contributed to the article and approved the submitted version.

## Funding

This work was supported by the National Natural Science Foundation of China (81970717, 82000740, and 82170845) and grants from the Key Research & Development Program of Jiangsu Province (BE2018742).

## Conflict of Interest

The authors declare that the research was conducted in the absence of any commercial or financial relationships that could be construed as a potential conflict of interest.

## Publisher’s Note

All claims expressed in this article are solely those of the authors and do not necessarily represent those of their affiliated organizations, or those of the publisher, the editors and the reviewers. Any product that may be evaluated in this article, or claim that may be made by its manufacturer, is not guaranteed or endorsed by the publisher.
