# Supplementary material for: Identification of Inflammation-Related Biomarkers in Diabetes of the Exocrine Pancreas With the Use of Weighted Gene Co-Expression Network Analysis
Source: Front Endocrinol (Lausanne). 2022 Apr 14;13:839865. doi: 10.3389/fendo.2022.839865 (PMC9046596; doi:10.3389/fendo.2022.839865)
Supplement: Supplementary file 1 [file DataSheet_1.zip › Additional files/Supplementary/Supplementary Table 2.docx]

| gene | logFC | P.Value | adj.P.Val |
| --- | --- | --- | --- |
| MMP7 | 2.10017456 | 0.000341333 | 0.00302145 |
| IGJ | 1.963472885 | 0.003347248 | 0.015205307 |
| GABRP | 1.865344636 | 0.000298558 | 0.002750334 |
| PLA2G7 | 1.837963485 | 0.00032612 | 0.002931637 |
| CEACAM6 | 1.815610119 | 0.01095175 | 0.03496221 |
| GLI1 | 1.670411823 | 6.60E-10 | 1.15E-07 |
| CXCL6 | 1.641813035 | 0.006076027 | 0.023210513 |
| CEP55 | 1.600347295 | 0.000497752 | 0.003902164 |
| LY86 | 1.57304253 | 9.19E-05 | 0.00113822 |
| BCL2A1 | 1.556838749 | 0.000631245 | 0.004625393 |
| IRF8 | 1.547217202 | 0.000144225 | 0.001617792 |
| CSTA | 1.539348609 | 0.001604175 | 0.008948045 |
| MICB | 1.539106526 | 0.000126131 | 0.001457993 |
| LY96 | 1.528042253 | 0.00217908 | 0.011185142 |
| VMP1 | 1.503339845 | 1.78E-06 | 5.97E-05 |
| ALDOB | 1.473466308 | 7.51E-07 | 2.99E-05 |
| NTS | 1.472090824 | 0.00197691 | 0.010424635 |
| RMI2 | 1.45595041 | 0.00019306 | 0.002002395 |
| ADAMDEC1 | 1.444074604 | 7.00E-05 | 0.000934304 |
| CD52 | 1.436653472 | 0.000585581 | 0.004380616 |
| LINC00951 | -0.921707539 | 7.53E-10 | 1.28E-07 |
| POMZP3 | -0.920164617 | 2.96E-05 | 0.000502866 |
| CYP2B7P | -0.766122425 | 1.53E-06 | 5.29E-05 |
| BTBD18 | -0.747868514 | 0.000667089 | 0.004817735 |
| GABRA2 | -0.718025797 | 0.002211627 | 0.011292708 |
| LOC100134368 | -0.711728557 | 2.18E-08 | 1.85E-06 |
| ISX | -0.690547673 | 0.000498497 | 0.003906258 |
| LINC00290 | -0.679142045 | 1.55E-05 | 0.000314578 |
| RASSF10 | -0.660956282 | 0.015416005 | 0.04471999 |
| MYOC | -0.654768666 | 0.002899771 | 0.013679807 |

**Supplementary table 1: The top 20 up-regulated genes and 10 down-regulated genes**
